# Food choices and practices during pregnancy of immigrant women with high-risk pregnancies in Canada: a pilot study

**DOI:** 10.1186/s12884-014-0370-6

**Published:** 2014-12-03

**Authors:** Gina MA Higginbottom, Helen Vallianatos, Joan Forgeron, Donna Gibbons, Fabiana Mamede, Rubina Barolia

**Affiliations:** University of Alberta, Faculty of Nursing, 3rd Floor Edmonton Clinic Health Academy, 11405 87th Avenue, Edmonton, Alberta T6G 1C9 Canada; Department of Anthropology, University of Alberta, 13-15 HM Tory Building, Edmonton, Alberta T6G 2H4 Canada; Alberta Health Services, Lois Hole Hospital for Women, 10240 Kingsway Avenue, Edmonton, Alberta T5H 3 V9 Canada; Department of Maternal-Infant and Public Health Nursing, University of São Paulo at Ribeirão Preto College of Nursing, São Paulo, Brazil

**Keywords:** Pregnancy, Food choices, Immigrant women, Perinatal

## Abstract

**Background:**

Immigrant women may be regarded as a vulnerable population with respect to access and navigation of maternity care services. They may encounter difficulties when accessing culturally safe and appropriate maternity care, which may be further exacerbated by language difficulties and discriminatory practices or attitudes. The project aimed to understand ethnocultural food and health practices and how these intersect in a particular social context of cultural adaptation and adjustment in order to improve the care-giving capacities of health practitioners working in multicultural perinatal clinics.

**Methods:**

This four-phase study employed a case study design allowing for multiple means of data collection and different units of analysis. Phase one consists of a scoping review of the literature. Phases two and three incorporate pictorial representations of food choices with semi-structured photo-elicited interviews. This study was undertaken at a Prenatal and Obstetric Clinic, in an urban Canadian city. In phase four, the research team will inform the development of culturally appropriate visual tools for health promotion.

**Results:**

Five themes were identified: (a) Perceptions of Health, (b) Social Support (c) Antenatal Foods (d) Postnatal Foods and (e) Role of Health Education. These themes provide practitioners with an understanding of the cultural differences that affect women’s dietary choices during pregnancy. The project identified building collaborations between practitioners and families of pregnant immigrant women to be of utmost importance in supporting healthy pregnancies, along with facilitating social support for pregnant and breastfeeding mothers.

**Conclusion:**

In a multicultural society that contemporary Canada is, it is challenging for health practitioners to understand various ethnocultural dietary norms and practices. Practitioners need to be aware of customary practices of the ethnocultural groups that they work with, while simultaneously recognizing the variation within—not everyone follows customary practices, individuals may pick and choose which customary guidelines they follow. What women choose to eat is also influenced by their own experiences, access to particular foods, socioeconomic status, family context, and so on.

The pilot study demonstrated the efficacy of the employed research strategies and we subsequently acquired funding for a national study.

## Background

Immigrant women may be regarded as vulnerable populations since challenges exist with respect to access and navigation of health services and more specifically maternity care services [1]. Difficulties may be encountered in terms of accessing culturally appropriate care in addition to other challenges such as language barriers and discriminatory policy and practices that may ultimately impact upon maternal health. Without culturally appropriate health care delivery a negative trajectory of events may occur that range from simple miscommunication to life-threatening incidents [2,3]. For this research, we utilize the Canadian Council of Refugees definition of an immigrant as a person who has settled permanently in another country. Immigrants choose to move whereas refugees are forced to flee [4]. Immigrant women are a diverse group that includes economic skilled workers, refugees and asylum seekers, and those without legal status [4].

### Diversity and health in Canada

Global migration contributes to increasing diversity within nation states resulting in diverse populations. Immigrant populations in Canada are more diverse than the Canadian-born population in terms of ethnic origin, first language, culture, traditions and socio-economic status [5]. As evidenced by the healthy immigrant effect [6,7], relatively healthy immigrants enter Canada, yet over time, many factors contribute to a decline in their health. This phenomenon largely affects those communities whose immigration is planned. Immigration effects include: health selection [6], acculturation and the stress of relocation that may erode health advantage [7], and distrust of Western medicine with a preference for seeking out traditional health care providers. However, populations who relocate as refugees or asylum seekers are found to have compromised health status, particularly women who are often traumatized by war, rape and the transgression of their human rights [8]. Many have lived in refugee camps for several years prior to immigration; this results in deterioration of their health status including those related to maternal health and maternity service provision. While substantial diversity exists within immigrant women populations, commonality may exist in terms of challenges encountered during access to and navigation of maternity care services and in experiences of migration, cultural adaptation and acculturation.

Without culturally appropriate health care delivery, a negative trajectory of events may occur ranging from simple miscommunication to life-threatening incidents [2,9]. Poor or inadequate initial health assessments and communications between the caregiver and patient may lead to unsatisfactory therapeutic encounters that in turn result in multiple consultations or failure to comply with treatment, thereby wasting time and resources for both patient and caregiver. There is currently a growing public health initiative which requires healthcare organizations to promote, protect and contribute to reducing health inequalities and culturally appropriate communication can be an important component to this aim.

### Maternal and birth outcomes of immigrant women: relevance of food choices and practices

Epidemiological research from Canada and elsewhere has reported equal or more favorable birth outcomes for migrants [10-13] supporting an “epidemiological paradox” associated with the concept of the “healthy migrant effect”. Numerous other reports highlight serious problems of equity in perinatal health outcomes [14-16] particularly for refugees [17] and other immigrants after increased lengths of stay (with the accompanying acculturation) [18,19]. A recent Canadian study found higher rates of low birth weight and full-term low birth weight (i.e., small for gestational age or SGA) for infants born to recent immigrant women [15] and immigrants living throughout Europe have been reported to be at substantial risk for pre-term delivery (24%), perinatal mortality (50%), and congenital malformations (61%) [14]. Hospital costs for preterm and SGA newborns are higher than those for their normal-growth counterparts by nine and two times, respectively [20].

Although there is no consensus that poor birth outcomes are associated with immigrant women, the potential negative health outcomes include significantly higher rates of gestational diabetes (predisposing the mothers to preeclampsia and type 2 diabetes and their offspring to obesity and type 2 diabetes) [21], low maternal weight gain (compromising both newborn and maternal health) [18], genetic anomalies such as neural tube defects due to lack of folic acid intake [22], and maternal anemia (increasing the risk of preterm delivery) [23]. All of these outcomes relate to food choices and practices, and thus food beliefs and behaviors could be important aspects in encouraging good health practices (and address any undesirable ones) to avoid poor health outcomes. Evidence suggests that everyday diets of immigrant women consist mainly of processed foods and animal proteins as well as foods high in fat, salt, or sugar [24,25]. It is reported that prevalence of obesity post-migration is widening because of the adoption of a Western diet [26]. Conversely, immigrant women reduce their dietary intake, even during reproduction, to maintain or quickly return to their hegemonic body ideals [18]. Successfully providing appropriate prenatal nutritional and diet education requires the legitimization of the pervasive traditional beliefs and practices of immigrant women [27,28]. Despite these observations, little attention has been paid to the food consumption and choices of immigrant women in the perinatal period and research is needed to elicit understanding of ethnocultural food choices and practices and to improve culturally based competency of maternity care.

### Study aim

The purpose of this project was to understand ethnocultural food and health practices and how these intersect in a particular social context of cultural adaptation and adjustment in order to improve the care-giving capacities of health practitioners (i.e. maternity nurses) working in a multicultural perinatal clinic located within a hospital. The funding stream awarded for this study has, as its ultimate aim, a goal of enhancing services in the hospital. As a secondary goal, this study aimed to assess the feasibility of the study design, and in turn expand the research with specific immigrant communities residing in different places.

## Methods

We employed a case study design that incorporated a participatory approach [29]. The case study is both descriptive and explanatory in nature and is appropriate to examine complex, real-life situations [29]. Our endeavour will acknowledge the complexity of everyday lives and acknowledge the existence of multiple realities. Decisions regarding health and illness prevention are fraught with ambiguity as individuals strive to maintain culture and traditions whilst integrating evolving modernities and the influence of globalization in daily existence. Food practices are an important component of marking individual and group identity. How an individual reproduces or resists normative practices is a means of understanding their social location. Migration, whether between countries or within Canada provides both opportunities and challenges for maternal health practices, including food practices. There is no question that social determinants affect approaches to meeting the human need for nutrients, but this research will provide a greater depth of understanding as to why and how such food practices develop.

Case studies also allow for multiple means of data collection, data collection in different settings, and different units of analysis. Our project was structured into four phases: **Phase One -** A scoping review of the literature; **Phase Two -** Pictorial representations of food choices (photovoice/photo-elicitation); **Phase Three** – Semi-structured photo-assisted narrative interviews of 10 immigrant women; **Phase Four -** Production of a culturally appropriate visual tool for immigrant women. The description of the methodological approaches of the all four phases is published, as are the findings from phase one [1,30]. In this paper we present a brief overview of the methodology and results of phases two and three.

### Phase one: scoping review

A scoping review was performed to examine and outline the extent, range, and nature of empirical evidence on immigrant and Aboriginal women’s food practices during pregnancy and childbirth. A framework for performing scoping reviews published by Arksey and O’Malley in 2005 [31] and Levac, Colquhoun and O’Brien [32] was used to conduct this review. This framework included: identifying the research question; identifying relevant studies; study selection; charting the data and collating, summarizing, and reporting the results. Further details on the methodology can be found in Higginbottom et al. [30].

### Phases two and three – photovoice and photo-assisted interviews

Photovoice is the process by which people identify, represent, and enhance their community through specific photographic technique [33]. This approach is particularly useful for individuals who speak English as an additional language. The camera as a research tool is well documented in disciplines such as anthropology and sociology [34-36]. Photography often leads to uncovering misconceptions and arriving at more reality-based understandings of phenomena [37]. A combination of photographs and accompanying narratives adds richness to data in qualitative studies. The technique also acknowledges that the participants’ perspectives are valuable and necessary to the understanding of a problem or event [38]. Through small or large group discussion, community members reflect on the images produced in a safe environment, and dialogues on potential solutions may emerge. Therefore, as a data collection method, photovoice serves the dual process of engaging communities on a topic of concern while providing valuable information about their current life in relation to a topic.

### Study setting: context of the study

Study participants were recruited from a Prenatal and Obstetric Clinic in Edmonton, Canada. In 2006, Edmonton’s population totaled one million, with 189,775 people identifying themselves as being foreign born [11]. The visible minority group totaled 174,729 (17%) largely being of Chinese, South Asian and Filipino origin [11]. Visible minority is a term used in official discourse in Canada, and speaks to the fact that minority groups with characteristics that are evident to others (e.g. physical traits) may have different everyday experiences (including potential discrimination) than those who are members of hidden minority groups (i.e. without traits marking their difference in the general population). South Asian is also used in official discourse and by local community organizations to include people whose ancestry originated in Pakistan, India, Nepal, Bhutan, the Maldives and Sri Lanka. The clinic in an area hospital was our recruitment site. This clinic provides consultation services for preconception counseling, prenatal screening, diagnosis, and treatment for women who are experiencing high risk conditions in pregnancy. Many of the high risk conditions, including obesity, diabetes, hypertension, intrauterine growth restriction, and oligohydramnios, are affected by women’s behaviors including food practices. The region surrounding this clinic is home to many immigrants. The population diversity of this area mirrors or exceeds the general population of Edmonton. Moreover, data in 2006 (latest available figures) indicated that interpretation services at the unit responded to 849 requests representing 33 languages. The reasons for requesting interpretation can be categorized in three broad groups: 1) to explain procedures during clinical examination, treatment or diagnostic tests; 2) to translate information about a diagnosis, treatment options, outcomes and other information for informed decision-making by the client with their health care provider[s]; and 3) to allow clients to communicate their perceptions of their condition, signs and symptoms, and general well-being to the physician, nurse or other health care provider. Since this time, interpretation services at the hospital have been outsourced.

Considering these language interpretation challenges and the needs for specialized education about nutrition during pregnancy at our study sites, a team was gathered in 2011 consisting of researchers from the university and clinicians at the hospital. A concern that emerged during these early meetings was the challenges maternity care nurses may face when eliciting and conveying information regarding optimum food choices during pregnancy because of language difficulties and cultural differences in reproductive health and food practices.

### Recruitment

Because of the partnership between the clinicians and researchers and the identified knowledge gap/issues faced by clinicians, and the funders’ focus on improving care and resources at the hospital, recruitment was focused at the clinic. This clinic receives approximately twenty requests for referral daily from local area doctors and obstetricians-gynaecologists (there are no self-referrals). A co-investigator (DG), or an informed intermediary who worked daily in the clinic, provided potential participants a brief description of the study and indicated where they could sign and submit (into a locked mailing box) a consent form allowing contact by the research team. From this point, contact information of the volunteers were referred to one of the investigators (GH or HV), or their designated research assistant, who would arrange for a meeting to gain informed written consent and initiate the photovoice and photo-assisted interview process.

### Sample population

A total of ten immigrant women were recruited. Participants were purposively selected to represent a range of migration experiences and ethnocultural communities. The inclusion criteria for immigrants were years of residence in Canada—participating women lived in Canada for at least two to four years—in the hope that they had become somewhat familiar with the Canadian health care system and had some familiarity with English if this was limited prior to arrival in Canada. Of the ten women, translators were used for two; in these cases, the women had some English skills, but felt more comfortable speaking in their native languages. A sample size of ten is adequate for a pilot study, and served to reveal general issues immigrant pregnant women face when adjusting their food practices to the local food environment. When this study was designed, we had also planned on conducting approximately ten interviews with Aboriginal women, because the clinical partners had identified the need for more information on cultural variation of food and health practices amongst the Aboriginal pregnant women using the hospital and clinic. Unfortunately, early in the recruitment process the Aboriginal coordinator at the hospital left the position, and this position was unfilled for months—the time period corresponding to data collection. Without a community collaborator who could navigate cultural and other issues, the team decided to not attempt to recruit Aboriginal women and to revive this aspect of the study at a more opportune time for all involved. Furthermore the research ethics framework in Canada demands a specific skill set including collaboration with Aboriginal community members [39].

### Data collection

After obtaining informed consent, a short interview of approximately thirty minutes using a topic guide was undertaken and then disposable cameras were provided to participants (the women received training on their use if necessary). Participants were asked to take photographs of all meals and snacks (including drinks) during a three-day period (including one weekend day) and other foods they perceived to be healthy/unhealthy for consumption during and after pregnancy. They were asked to hand in their camera to the Clinical Nurse Specialist at their next visit to the clinic, who in turn handed them over to the PIs for development. Subsequently, the PIs or a research assistant conducted a semi-structured narrative photo-assisted interview, where each woman was asked to tell their story through the photos, to discuss whether food choices represented are typical or not, what factors influences their dietary choices, and what they would like to change. This revealed not just what women are typically eating but the kinds of everyday issues that influence their food practices. Some questions addressed culturally normative practices surrounding maternal food choices and consumption, including how women negotiate normative practices within their own worldview and experiences. A methodological approach of photovoice known as photo-elicitation was also used to complement interviews and better attain an understanding of taken-for-granted beliefs and assumptions about food practices during pregnancy and the postnatal period. Total time commitment for the each participant was about four hours; they were given a small honorarium in appreciation. The pilot study was focused on the hospital partner setting and consequently we focused on the users of the facility. We were looking for common experiences of immigrant women; however we recognize that there is heterogeneity within and between various immigrant communities. The large-scale national study that followed this pilot is investigating variations in women’s experiences [40].

### Data management & analysis

Data was stored, managed, classified and ordered with the aid of Atlas-Ti, a qualitative data analysis software package. Atlas-Ti is useful for this study as the software package facilitates analysis of visual representations. The process of analysis is characterized by identification and classification of data and progresses to abstract generalizations and explaining patterns - it is not linear but undulating and cyclical. We drew upon the analytic framework of Miles and Huberman [41], which includes: 1) Familiarization with the transcript, 2) Identification of open and in vivo codes, 3) Utilization of both theoretical and commentary memos, 4) Funneling and rationalization of redundant codes, 5) Creation of themes categories and families, 6) Creation of graphic network views in Atlas-Ti demonstrating relationships between the various codes and categories, 7) Constant interrogation of the data and the challenging of initial assumptions, 8) Identification of outliers and non-confirming data, 9) Reflective team meetings to achieve higher level of abstraction in the analysis, 10) Creation of hierarchies, classifications and typologies, and 11) Creation of a written narrative. This characterizes the iterative process associated with qualitative data analysis as preliminary interpretations are challenged and data are revisited in the light of further data collections and new insights into the data.

### Ethical considerations

Before commencing research, ethics approval was obtained from the University of Alberta Health Research Ethics Board and operational approval was obtained from the Prenatal and Obstetric Clinic and the partner hospital. Voluntary written informed consent was obtained from all participants. As part of the consenting process, participants were assured that they did not have to answer every question, could choose to be audio-recorded (or not), and could withdraw at any time. To ensure that participants were fully informed, a translator was used for both the consenting and interviewing processes when necessary. The principles of informed consent, confidentiality and anonymity were observed at all times (including storage of materials).

## Results

### Phase One

Detailed description of the scoping review and findings are published elsewhere [30] consequently we briefly summarize the major themes of the review here to compare themes with those revealed from our interviews. All qualitative studies highlighted the importance of understanding the diversity in cultural practices of immigrant pregnant women when promoting healthy pregnancy outcomes. Major themes included: 1) *Cultural practices and beliefs regarding the perinatal period*: Studies identified the importance of dietary or food-related cultural practices during pregnancy. Topics included dietary practices, religious rituals, values and beliefs related to the fetus, maternal health, and the role of family members and support groups during pregnancy. 2) *Family and social support during pregnancy and delivery*: A major challenge for immigrant pregnant women can be the roles played by partners, family members, and social supports during pregnancy, especially since often immigrant women are away from their extended family and communities [22,42-44]. Immigrant women expressed feelings of loneliness and missed the social support which they could receive in their own countries. Pregnant women mainly received support from their husbands. 3) *Healthy pregnancy*: While all studies reviewed described the importance of healthy foods and emotional wellbeing to experience a healthy pregnancy, a few participants reported craving harmful substances, such as alcohol, and stated that one must be careful not to drink too much because it is harmful for the baby [45]. These food cravings were viewed by the women to be part of a healthy pregnancy and their consumption thought good for their babies [46,47]. 4) *Weight gain issues*: Overall, immigrant and Aboriginal women were concerned about the weight and adequate growth of their baby. However, a few immigrant women were purposefully not eating enough because they believed that too much food would result in a large baby and hinder a vaginal birth. On the other hand few immigrant women felt that need to eat more when they are pregnant because “they’re eating for two now” [47:202], some of them believed that mother weight gain will reflect the baby’s’ weight [47].

5) *Concern for health of the baby*: Immigrant women were more worried about their unborn child’s well-being than their own health. The ‘important’ thing for women was to have a healthy baby, and they were prepared to modify their lifestyle [47]. 6) *Specific food items*. Immigrant women also categorized the specific food item into “good” or “bad” food during pregnancy. Good food includes foods such as fruits and vegetables and bad foods include oily and fatty foods. Low income and the higher cost of healthy foods were identified as major barriers to healthy eating identified by these women [46]. Three major themes derived from the quantitative studies included in the review were nutritional assessment, cultural practices, and seafood consumption and other environmental exposures in the diet. While the first two themes highlighted similar issues raised in the qualitative studies, the latter theme focused on potential toxic contaminants in foods, such as heavy metals in seafood. The review indicates that there is a complex interplay of cultural, social, and economic factors affecting food choices and nutritional adequacy for pregnant immigrant women. As a result, influencing food and health behaviors may be particularly difficult in groups where the recommended changes are perceived to clash with one’s own customs.

### Phases two and three – photovoice and photo-assisted interviews

The ten participants in this study were immigrant women who migrated to Canada from a range of regions in Africa and Asia, however most (6) had come from Asia, predominantly from South Asia. Three participants reported being employed, and only one of these worked in a profession in which she had prior experience. All participants were married, with an average age of 33.5 years, gravida range of two to four, and an average of 1.6 children. Only three participants had previously given birth in Canada. Women who have larger numbers of children and thus a greater range of prior reproductive experiences to draw upon may have different food practices or adherence to ethnocultural food practices than those reported here. However this sample does provide a snapshot of relatively recent immigrant women’s experiences, who had little prior exposure to the Canadian health care system and reproductive health (including nutritional) practices.

The study was constructed so that women’s ideals and perceptions on healthy dietary practices during pregnancy would be discussed in the first interview, while in the second interview, through discussions of the photographs each woman took, a better understanding of actual practices and everyday challenges would emerge. Interestingly, the same themes emerged in the independent analysis of the first and second interviews, which suggests these themes are very salient for the participating women. Analysis of the interviews revealed five themes.

#### Perceptions of health

This theme incorporates participants’ beliefs on physical health, emotional/psychological health, and nutritional health. Some participants focused on a specific indicator, while others used a combination of factors with varying degrees of importance placed on each. The meanings attached to health affected what women ate before, during, and after pregnancy as well as behaviors and practices unrelated to food choices, such as exercise or attempting to maintain a positive mental outlook. This includes perceptions of what makes a person healthy in general, as well as the pregnant body specifically, and is illustrated with the following quotes:

*Okay, eat healthy and the other thing, walk during when you are pregnant, not be very lazy, like lie down all the time and rest. That is not good during the pregnancy. Drink milk, take your protein and everything which is useful for the baby*. [17:1]

*Like being healthy in pregnancy eating well, sleeping, sleeping well…you can feel your baby moving, you go to the hospital, they check you and say baby’s okay… But for pregnant women I think we have to take all the veggies, yeah, and the banku and then the fish. Fish and meat is also very good for us*. [8]

Healthy foods are not just types of foods, such as “veggies,” “proteins” or “milk”, but also reflect the quality of foods, as illustrated by one participant’s description of the importance of “fresh” foods: “*Very important. Fresh, not stay overnight, the one-week old, two days old. Every time my mom give me fresh food and homemade. Don’t eat outside a lot”* [4:2].

**Figure 1:**
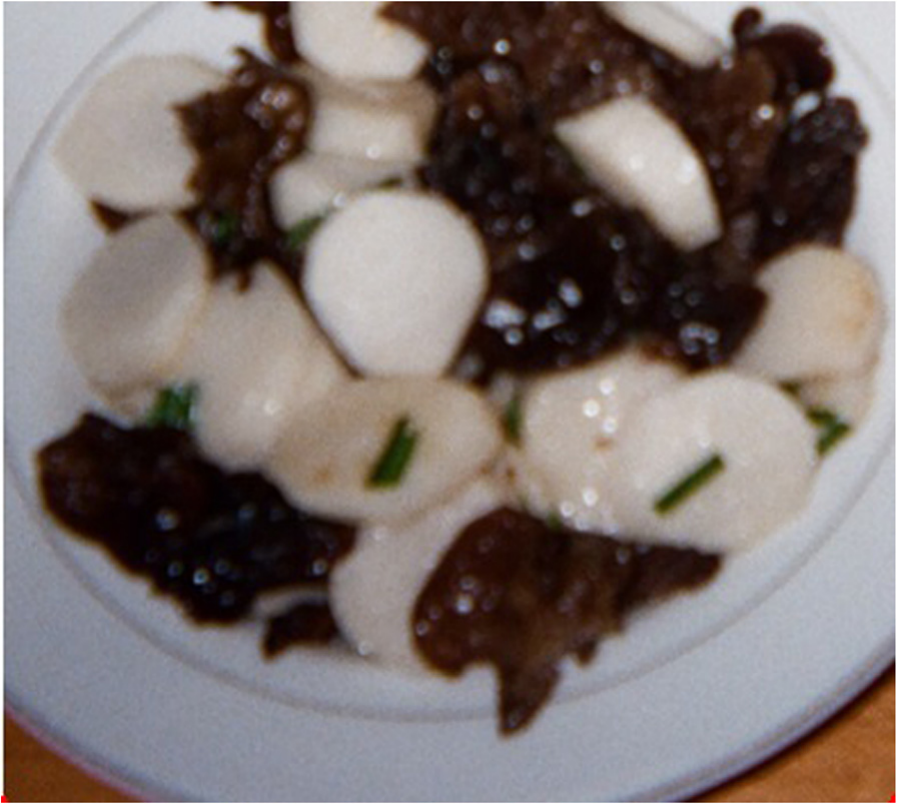
**Illustrates how one participants cultural beliefs about colour of foods affected concepts of healthy pregnancy foods.**

The following image (Figure 1) was taken by one participant to illustrate how cultural beliefs of healthy foods affects her everyday eating behaviors. Important here is not just what the food items are, but the combination of foods and their colors: “*in Chinese culture, people think the black food, red food, are good for our body. Yeah, especially for the blood”* [27:2].

Considerations of how food choices and behavioral practices can affect the health of one’s developing child also affected the health and dietary practices of mothers, and these understandings of how (un)healthy bodies were maintained or developed are partly informed by customary cultural knowledge and practices.

#### Social support (family & community)

The role of family, partners, friends, and community members in supporting and enforcing healthy eating and healthy behaviors were highlighted by almost all immigrant women. There were a variety of kinds of support described by participants, including those related to pragmatic everyday activities (e.g. housework, cooking, childcare), as explained by one woman, “*Yeah. After delivering baby at least two or three week, traditionally the family support everything. I don’t need to care about the housework; I don’t wash dishes, laundry, anything. Just stay relaxed, take care of your baby*” [4:2]. Other women described the intangible emotional encouragement that contributes to the support they receive from others, “*When if it’s – let’s say if it’s your first pregnancy everybody like, won’t let you get down from the bed. … they will be very careful for you”* [15:1]. There was a wide variety of experiences within this theme, influenced by each woman’s cultural background, socioeconomic status, and access to support networks. Many had relatives living nearby, so there was at least one adult the women could rely on, as one woman noted, “*And if my mom is not here, maybe husband prepare everything. … even if he can’t do all but he will have to help something and then leave while these things were okay”* [4:2].

Some women’s support networks were stretched across space. This is exemplified by women receiving advice from relatives over the telephone or internet. For example, one woman from western Africa spoke with her mom regularly via phone and Skype, noting that: “*No, she tell me every day, “Take care of you, don’t work too much, don’t do something you don’t like it (?).” I say, “Okay, mom.” I said, “Okay, mom.” She’s scared because I’m here just with myself. My husband is not at home sometimes and I have another two boys and this is not easy*” [14:1]. Based on the importance of the social support networks described by the women, it suggests that for those without family or friends in Canada or who were unable to speak English, social support may be low or absent altogether. The absence of social support for antenatal or postnatal women can be a significant factor in their antenatal and postnatal choices, behaviors and overall health status.

#### Antenatal food choices

This relates to the foods consumed and avoided before and/or during pregnancy. These choices correlated with cultural beliefs on what a healthy pregnancy entailed, advice given by friends and family, and past pregnancy experiences, as exemplified by: “*During pregnancy, they said that during pregnancy you should drink as much as you can, so like it makes your uterus like really strong and it makes it like, let’s say slippery so the baby can just slip down, slip down easily*” [15:1]. This South Asian woman, pregnant for the second time, heeded the advice of family, despite having lived in Canada for ten years. Thus, not only biomedical health care providers, but also family, are important sources of dietary knowledge.

In some cases, pregnant women continued eating similar foods to what family members consumed, as one participant explained, “*We were all eating the same thing, the pita, and chapatti during pregnancy*” [20:2]. However, in most cases, pregnant women ate more frequently, consumed specific foods thought to improve birth outcomes, and/or ate greater quantities. For example, one South Asian participant [22:2] noted that pregnant women were encouraged to have butter in every meal. “*Butter, butter, butter; every meal butter. Even during pregnancy, like after seventh or eighth month, they said if you eat more butter it will soothe all your birth canal so the delivery will be easier.*” This echoes the South Asian woman’s comment on the importance of drinking liquids above, so as to improve ease of delivery. Clearly, these ideas are linked to women’s culturally informed concepts of the body, and how bodies work.

Some cultural ideas of food and bodies are based on the concept of balance, originating in various humoral medical systems. For instance, pregnancy in many humoral medical systems is viewed as a ‘hot’ condition, thus ‘hot’ foods must be avoided. This is reversed after birth, where ‘hot’ foods are consumed. This notion of balance is alluded to by this woman, “*And I was not allowed to drink water out of the tap because it was cold, so they always give you warm water to drink, you know, for fear of catching a cold. That’s one thing. But of course, you know, I cannot have water without ice so I’m so used to the cold water*” [20:2].

Other cultural ideas on the body also impact food practices. For example, a participant described how pregnant women were advised to drink a lot of milk (and apples too) so that the baby would be fair-skinned. This was important not only for aesthetic preferences, but also for the health and wellness of the child: “‘*white’ or have a lighter skin color, and lighter baby signified a healthy baby: the baby will be whiter, the baby. Because the skin color will be lighter, your baby will be healthy. But the milk especially, they - every people, apples and milk”* [22:2].

Food cravings were another common experience shaping pregnant women’s food choices. Food cravings may pose a dilemma for pregnant women, in terms of whether to succumb or resist the craving, especially if the food they were craving was viewed as unsafe or unhealthy during pregnancy. For example, one South Asian woman admitted that while “*People say caffeine is not good during pregnancy…. I know it’s not good but I just couldn’t, you know, give it up. So I just have to*” [20:2]. This participant also noted the common experience of craving familiar foods of home:

*“Usually, you know, in India when women are pregnant people do go ahead and make special pickles at home because they have the craving. You know sometimes you just want to go and have pickles. Pickles in the sense that yeah, even here, you know, in North America people do want to eat pickles, the bottled ones, you know, the dill or whatever you call them, you know. …they crave for this pickle because it’s a little sour and spicy*”.

Food cravings cam become a complex matter for women with an underlying health condition such as gestational diabetes, as one participant [22:2] described; “*So craving is something. [laughter] But the diabetes was a very big issue. Like my readings go very high during pregnancy, my hormones changes so much. For some people, my doctor was saying, for some people it’s not that bad, but for some people they just mess up all the hormones and mine was one of those*.”

**Figure 2:**
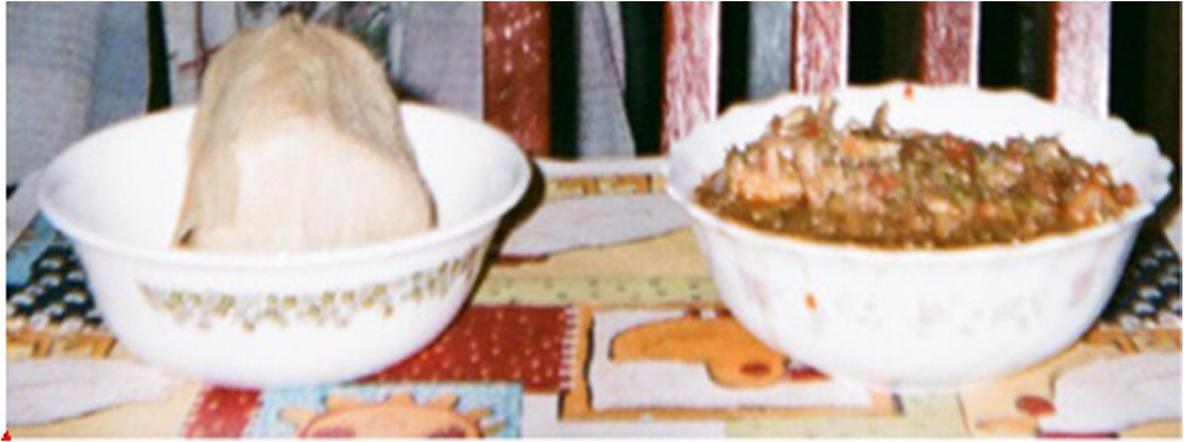
**Illustrates how one participant’s food aversions were mediated through culturally informed practices.**

Food aversions are also mediated through culturally informed practices. One participant illustrated this through the following image (Figure 2),

“*noting: Yeah, but in our context when we say - let’s say somebody is going to eat banku and pepper, it means you add pepper with tomatoes, with onions, or with ginger and garlic or whatever. But ginger and garlic are optional, so we take pepper, onions and tomatoes, add a little salt to it. You grind them together and then take it with banku, yeah, just to kill the nausea.”* [8:2]

The ethno-cultural socialization and experiences of women highly influenced their antenatal behaviors, especially in terms of food consumption patterns. For some women, religious beliefs or health challenges limited their food choices, although most seemed aware of possible dietary deficiencies and had alternatives in place. Socioeconomic status was variable among the sample and was not explicitly stated to be an issue affecting the ability to make healthy choices; however, it is worth considering that lower socioeconomic status affects one’s ability to purchase nutritionally dense foods. Geographical location was mentioned by some participants as a factor in their ability to purchase certain seasonal foods, or foods not readily available in Canada as they are in their home countries.

In addition, at least one participant mentioned the urban/rural location distinction affecting dietary choices. In terms of availability, the existence of specialty ethnic food stores or aisles in supermarkets allowed most women to find cultural and religiously sanctioned foods, however, some foods were simply not available or too expensive in Canada. Lastly, all of the women in the sample were experiencing high-risk pregnancies, and so they were dealing with previous or developed medical conditions (such as gestational diabetes) that affected their choices and behaviors.

#### Postnatal food choices

Immigrant women were very concerned about the foods that are consumed or avoided directly after and in the forty days following birth. These choices correlate with cultural beliefs surrounding health and healing, production of breast milk, and advice from social networks. For instance, “*you eat some sweets, the sweets will give you milk for the baby, and nuts. And we drink like hot tea with cinnamon, cinnamon with ginger for cleaning the blood after delivering the baby”* [7:1]. Following such beliefs often depended on having the social support, typically of a female kin, who would prepare these foods, teach which foods ought to be consumed in the forty days post-birth, and who could ensure the new mother rested and followed these post-birth food traditions (see social support theme). Women that had previous children referred back to those experiences when making choices regarding food practices, and her educational status was an important factor in how post-birth food traditions were adhered to.

**Figure 3:**
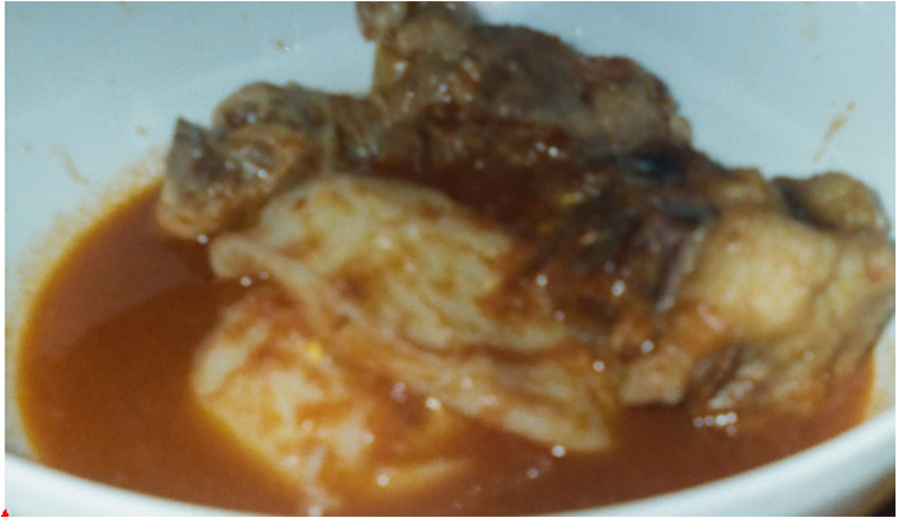
**Illustrates immediate postpartum food choices that one participant’s female kin prepared for her.**

The immediate postpartum food choices and practices depended on the type and place of delivery. For medically assisted delivery (i.e. Caesarean section), the doctor prescribed the meals for the first few days. However, for a normal vaginal delivery in a hospital, regular light, semi-liquid, energy rich foods were provided. But many women also described the foods their female kin would prepare: “*So the palm fufu, if it’s in the afternoon your mommy will bring fufu and soup and then you take for you to replenish your energy that you spend during bearing [child], and then the soup itself, you will drink it, for you to get enough milk, yeah, to come*” [8:2] (see Figure 3)

.

**Figure 4:**
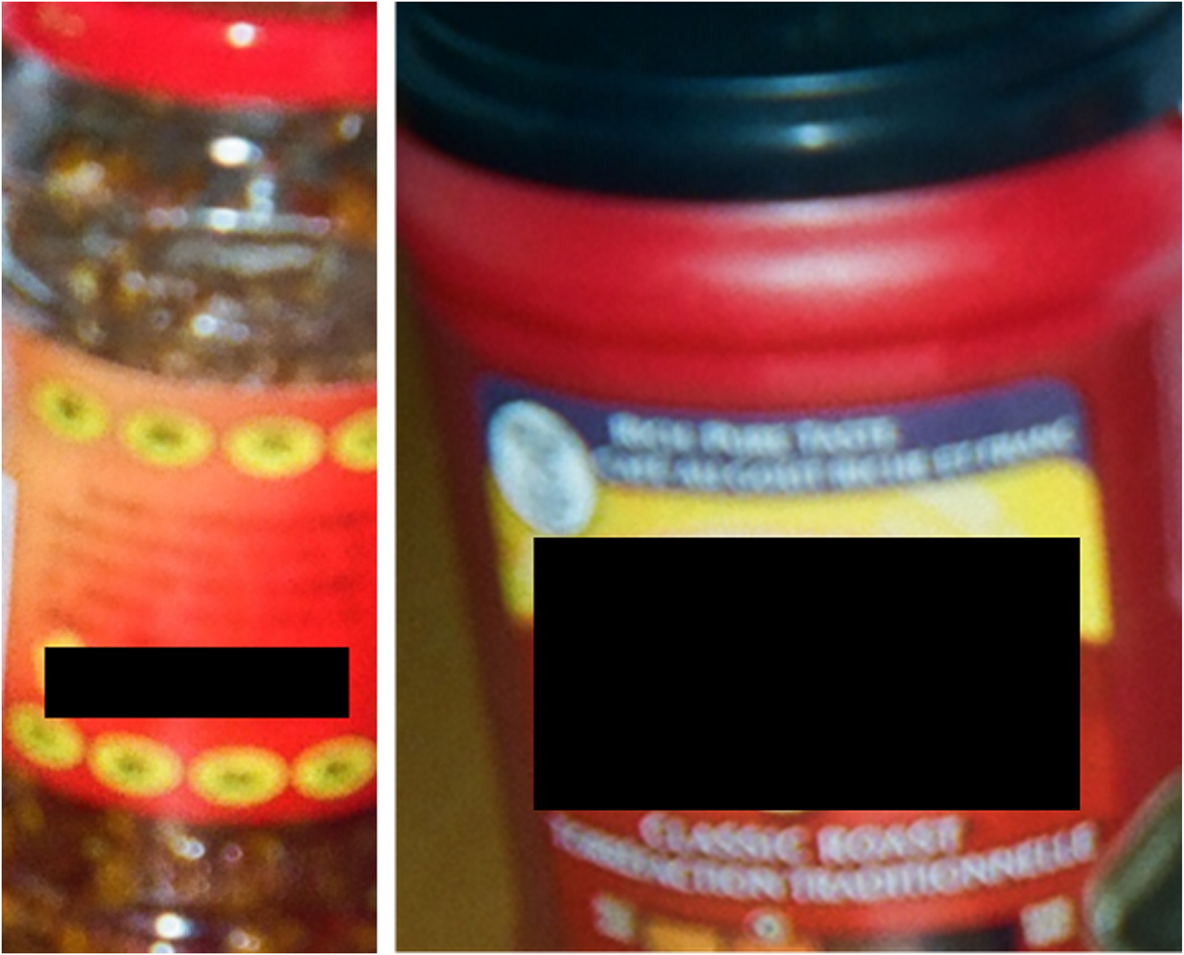
**Illustrates foods that one participant avoided while breastfeeding.**

Breastfeeding affected women’s food consumption practices, dictating the kinds of foods that would foster milk production, but also be healthy and nourishing for the newborn’s sensitive stomach: “*Because they said if you eat more of those starchy foods they will make you more gassy and the baby will be more uncomfortable, so try to avoid starchy food - rice, more pitas. Try to fill your stomach with more proteins, even though nuts or whatever you can”* [22:2]. Another participant provided images (Figure 4) of foods she avoided while breastfeeding, explaining: “*I breast feed my baby, that’s why also I try to avoid any alcohol and coffee and spicy food … stay as much as I can stay healthy, you know. Don’t eat any junk food and not the fat, everything, you know. For my health, also for my baby’s health. … If I eat any, you know the unhealthy food, baby is going to be, you know, take unhealthy milk from me. [laughs]”* [4:2].

#### Role of health education

**Figure 5:**
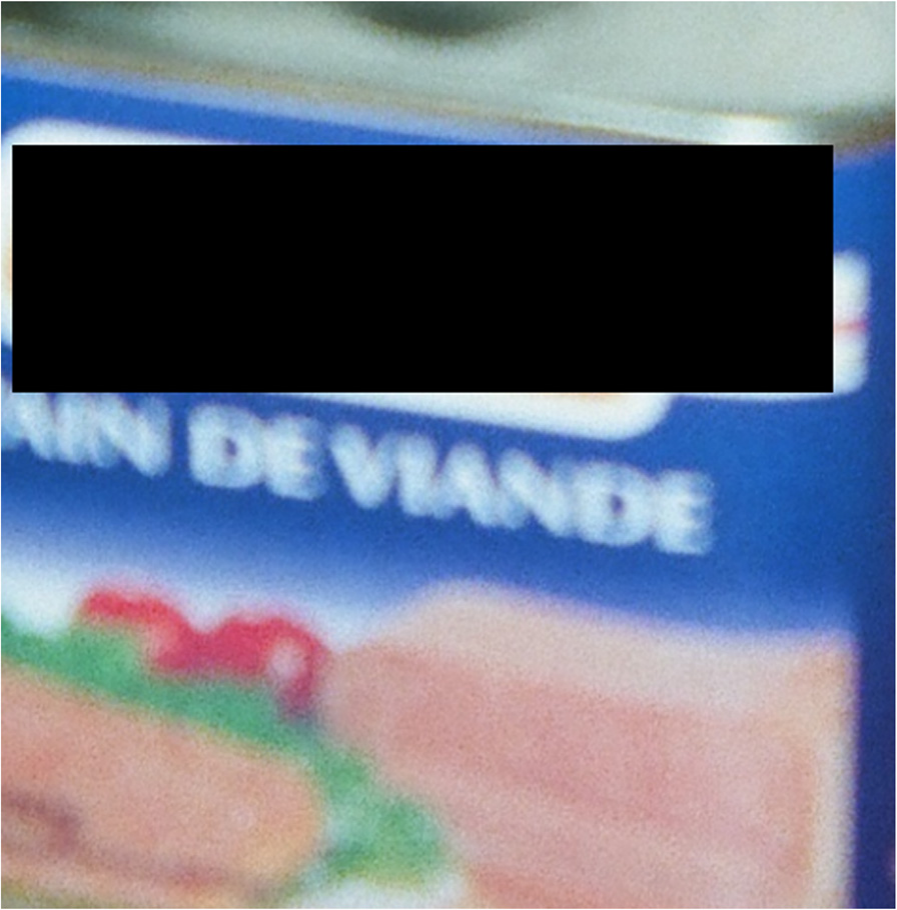
**Illustrates food that one participant was advised by a dietician to avoid.**

We also examined the sources of information and knowledge on healthy eating during pregnancy. Medical doctors were a critical knowledge source, as illustrated here: “*Before I eat it because I like hot sauce and I like chicken wings. Yum, so good. [laughter] But this doctor is saying that’s not very good for a baby”* [14:1]. Another women showed the following image (Figure 5) to explain, “*I want to show you, this food we are trying to avoid. … Yeah, I heard it from my dietitian. I never heard of this before, actually. I didn’t know*” [27:2].

Another woman shared, “*they tell me that don’t eat so much cold things like cold water; those are not good. … We avoid yogurt. … And they told me avoid rice too. … But according to doctors, rice are good, I think so. That’s why they are in their manual, give her rice”* [17:2]. This example illustrates how biomedical and customary advice may sometimes be at odds, and women must make their own decisions on which recommendation should be followed. Thus, women incorporated other knowledge sources as well, such as family members, friends, community members, media, and their own experiential knowledge (where relevant). One woman noted*, “mom taught me like that and, yeah, she’s teaching me like that. Also, I am going to do [i.e. teach] my daughter, yeah*” [4:2]. Women’s relatives are frequently the first source of advice and ministrations, but many women also rely on their experiential knowledge, as illustrated in the following exchange:

*P: I would say all of them, because that was the food I was eating.*

*I: Why do you feel that those foods are healthy while you’re pregnant?*

*P: Because I’ve been eating [these foods] since I was born so I don’t see anything [wrong]*. [10:2]

The foods this woman had consumed for her entire life could not possibly be unhealthy, for in this woman’s opinion, she would have previously suffered illness if that was the case. This is an important consideration for health care professionals, to navigate cultural norms and individual experiences when advising on healthy dietary practices.

Because all participants were experiencing high-risk pregnancies and dealing with a variety of health issues, many referenced these experiences when explaining their food choices.

Through the process of immigration, some of the women exhibited acculturation in the food choices and in the acceptance of Western biomedical understandings of pregnancy. Furthermore, some women decided not to adhere to “traditional” guidelines. However, the women in this sample largely favored advice given by family members, continuing to follow customary food practices of their foremothers.

## Discussion

The scoping literature review emphasizes the limited amount of data on this topic. More studies are required to elicit deeper understanding of ethnocultural food choices and practices, in order to fully inform processes to improve cultural competency of maternity care. Therefore our pilot study using photovoice and photo-assisted interviews had provided deeper understandings of ethnocultural food choices and practices. Our findings from the photo-assisted interviews validates the finding of the scoping review that there is a complex interplay of cultural, social, and economic factors exists to affect the food choices, intake and nutritional adequacy for pregnant immigrant women [33].

Family and social support during pregnancy was important for participants, especially when they are not with their extended family post-migration, as has previously been reported [22,43]. Immigrant women in our study reported various experiences of how much they can rely on support network during pregnancy [42]. Antenatal and postnatal food choices of these women also confirmed that the meaning of food in a particular life event such as pregnancy could not be changed even when these women were living in the country of migration for longer period of time [48,49]. Immigrant women may adhere to certain cultural food beliefs and practices more rigidly than their friends and family that have remained at home [14]. As a result, influencing dietary behavior change may be particularly difficult in these groups, particularly when the changes are perceived to clash with one’s own customs that provide emotional connections to home. It is important to note that these cultural food items may be more important in some families and communities than in others, and the existence of intracultural diversity—further research is required on factors affecting dietary change amongst immigrant pregnant women, and the heterogeneity of practices within various ethnocultural immigrant communities.

The role of health practitioners, especially medical doctors were identified as an important source of information in our study. One possible reason we found health personnel as important sources of dietary information is the location and context of the study. The clinic is recognized for providing language interpretative services for diverse cultural groups. Conversely a few studies have reported that immigrant women experience difficulty in following healthy diet advice during pregnancy that is provided by health care personnel. Some studies indicate this is most challenging when they are from low-income groups [42,45,50], although cost of food was not as salient for our participants. We suggest that this may be due to the fact that most of our participants (seven) were not employed outside the home, and were cooking traditional foods prepared with basic ingredients. For example, it is cheaper to purchase dried beans, grains, and so on than to buy processed or partially prepared products. Nevertheless, we recommend that clinicians may need to spend additional time discussing dietary difficulties with their patients when cost of food impacts accessibility. Additionally, nurses, physicians and dietitians may benefit from discussing lifestyle factors with the pregnant women’s entire family because pregnant immigrant women may not make food decisions in isolation; their husbands or elder female kin may bear responsibility for family food decisions and practices.

Our study provides a snapshot of relatively recent immigrant women’s experiences, which had little prior exposure to the Canadian health care system and reproductive health (including nutritional) practices. The thematic analysis emphasizes the importance of local social milieus of immigrant women in providing the context for healthy practices and behaviors. Sources of nutritional knowledge include female family members, but also health practitioners. This is an opportunity to incorporate cross-cultural normative practices when advising immigrant women on health dietary practices during pregnancy. We suggest that honoring and respecting traditional knowledge will potentially make it easier to explain biomedical dietary norms and recommendations.

Based on our findings, we recommended that clinicians working within our hospital partners’ setting: (a) look for intersections in cultural food practices during pregnancy with biomedical recommendations and build upon these when providing dietary advice; (b) infrastructure and policy activities of the hospital should continue building collaborations with various ethnocultural communities beyond language translation services to support health pregnancies, recognizing the particular constraints and opportunities in various immigrant communities (e.g. community outreach by hospital dieticians may be useful); and, (c) Health care policies of the hospital need to provide support for practitioners, particularly with respect to cultural knowledge they may need to improve their effectiveness, perhaps during some of the regular training sessions.

### Study limitations

Recruitment from the diverse ethnocultural group was the key challenges in this research project. To recruit women for the study, we relied on our hospital partners. Although the study was initially conceived to include Aboriginal pregnant women, this was not done because the Aboriginal hospital coordinator left the position and the team decided that without an Aboriginal coordinator/liaison it was not appropriate to proceed. Although our hospital partners working with immigrant women were enthusiastic, recruitment proved to be time-consuming, and recruitment involved additional time constraints on our partners’ schedules. To assist with recruitment, a brief information sheet with permission to contact (i.e. potential participants gave consent for researchers to contact them) was developed. It also seemed that women were often in the latter part of their pregnancies when recruited, and in a few cases this meant that they were no longer pregnant by the time they were contacted. Furthermore, the hospital setting arguably had stressful connotations for potential participants which may have influenced their willingness to participate. Consequently, of the twenty-one women who indicated that they would be willing to be contacted, only ten agreed to participate in the pilot study. Clearly potential and actual participants had many stresses to grapple with and participation in this research study, even for those who were interested, was not always possible. A high-risk pregnancy is stressful for anyone, but particularly so for those who may have cultural and/or linguistic barriers and limited social support networks.

Our study sample consisted of women mostly from South Asia (six out of ten). Among our participants, shared dietary experiences when pregnant post-migration centered on the importance of support networks for pragmatic and emotional support while pregnant, and as knowledge sources for appropriate dietary practices (e.g. ethnocultural beliefs on appropriate foods to consume or avoid during pregnancy). Although this seemed salient for all our participants, it ought to be kept in mind that immigrants from other places, particularly Latin America which was not represented in our sample, may have other factors shaping food beliefs and practices in pregnancy and in the immediate post-natal period. Furthermore, all the participants were experiencing high-risk pregnancies, and this may have influenced our results. Nevertheless, findings of this pilot study did enable us to progress to a large national study, in which we are currently examining immigrant pregnant women’s food beliefs and practices from select ethnocultural groups, to better understand variations in beliefs and practices between and within specific immigrant communities [40].

## Conclusion

In a multicultural society that contemporary Canada is, it is challenging for health practitioners to understand various ethnocultural dietary norms and practices. Practitioners need to be aware of customary practices of the ethnocultural groups that they work with, while simultaneously recognizing the variation within—not everyone follows customary practices, or individuals may pick and choose which customary guidelines they follow. It is critical that health care providers recognize the complexity in cultural food and health beliefs and practices, and how socioeconomic status, age, ethnicity and family structure and support may differentially shape these beliefs and practices. Findings of this project are valuable for health practitioners to recognize that not all women accept customary dietary rules. What women choose to eat is also influenced by their own experiences, their access to particular foods, their socioeconomic status, family context, and so on. Thus, it is important to not assume that all people of a particular cultural group share the same dietary notions and food practices. The project team suggest that building collaborations between practitioners and communities to support healthy pregnancies and social support for pregnant and breastfeeding mothers is of prime importance. Working with community organizations will also be important in continuing to develop culturally appropriate dietary recommendations that incorporate biomedical knowledge and customary practices. The pilot study enabled us to establish the feasibility of our approach in terms of methodology and recruitment strategies for a larger study; in particular, we realized that recruiting at the perinatal clinic was not the best approach, so for the larger study we partnered with community groups for recruitment of immigrant women with normal pregnancies. For our hospital partner, we recommended that policies and practices ought to consider cultural food practices, building on traditional strengths while addressing customs that may not be ideal for the health of the mother or child. It cannot, though, be forgotten that additional approaches such as social and financial assistance programs addressing the array of health determinants are also required. These findings may be useful in other clinical settings providing care for immigrant women.
